# Dioscin Attenuates Myocardial Ischemic/Reperfusion-Induced Cardiac Dysfunction through Suppression of Reactive Oxygen Species

**DOI:** 10.1155/2021/3766919

**Published:** 2021-10-05

**Authors:** Dayin Lyu, Qing Tian, Huitao Qian, Chang He, Tianyu Shen, Jinxing Xi, Pingxi Xiao, Qiulun Lu

**Affiliations:** ^1^Key Laboratory of Cardiovascular and Cerebrovascular Medicine, Collaborative Innovation Center for Cardiovascular Disease Translational Medicine, Nanjing Medical University, Nanjing 211166, China; ^2^Intensive Care Unit of Wuhan Asia Heart Hospital, Wuhan 430000, China; ^3^The First Clinical Medical College of Nanjing Medical University, Nanjing 211166, China; ^4^Department of Orthopedics Surgery, Taihe Hospital, Hubei University of Medicine, Shiyan 442000, China; ^5^Nanjing Vocational College of Information Technology, Nanjing 211166, China; ^6^Cardiac Department, Sir Runrun Hospital Affiliated to Nanjing Medical University, Nanjing 211166, China

## Abstract

Myocardial ischemic/reperfusion (MI/R) is a leading cause of cardiovascular disease with high morbidity and mortality. However, the mechanisms underlying pathological reperfusion remain obscure. In this study, we found that dioscin, a natural product, could be a potential candidate for treating MI/R through modulating cardiac dysfunction. Mechanistically, our work revealed that dioscin could suppress the production of reactive oxygen species (ROS) *via* repressing the nicotinamide adenine dinucleotide phosphate (NADPH) oxidase 2 (*Nox2*) and enhancing the expression of antioxidant enzymes, including superoxide dismutase (SOD), catalase (CAT), glutathione (GSH), and glutathione peroxidase (GPx). These findings indicate that dioscin may be a potential candidate for therapeutic interventions in MI/R injury.

## 1. Introduction

Myocardial ischemic/reperfusion (MI/R), a leading cause of cardiovascular disease with high morbidity and mortality, is caused by the blood recovery after a vital period of coronary artery occlusion, which easily leads to myocardial infarction or heart failure [1–3]. Previously, literature reported that lethal reperfusion injury accounts for up to 50% of the final myocardial infarct size [4]. It is well known that the pathogenesis of MI/R injury includes oxidative stress, inflammatory response, calcium overload, and mitochondrial dysfunction [5–8]. Since the oxidant stress is accompanied with the pathological process of cardiac dysfunction, inhibition of oxidant stress is a potential therapeutic strategy for MI/R injury [9].

Oxidative stress is originated from the overwhelmed reactive oxygen species (ROS) and the insufficient antioxidant defense systems [10]. Under the physiological condition, ROS is crucial and maintains normal cellular metabolism processes, balancing at the dynamic stage between antioxidants and oxidant response [11]. However, abundant of ROS is generated in two stages of MI/R, including ischemia and reperfusion [12]. Due to sudden overburden of the high oxygen tensions, reperfusion contributes to elevated levels of oxygen free radical (OFR) production, which leads to oxidative damage, such as protein carbonylation and DNA oxidation [13, 14]. Nevertheless, various traditional antioxidants do not present with therapeutic efficacy [15, 16]. Some active natural products, including pentamethylquercetin, isorhychophylline, myricetin, and fisetin from medicinal plants, have shown excellent activities against MI/R injury [17–20]. Thus, it is reasonable to exploit effective natural products from herbs for the treatment of MI/R injury.

Dioscin, a natural steroid saponin isolated from the root bark of wild dioscorea nipponica, is currently widely used for cardiovascular disease treatment [21]. Our previous investigations have demonstrated that dioscin elevates lncRNA MANTIS in therapeutic angiogenesis for myocardial infarction [22]. What is more, dioscin plays a beneficial role in hepatic ischemia-reperfusion injury, intestinal ischemia-reperfusion injury, and gastric ischemia-reperfusion injury [23–25]. However, whether dioscin protects cardiac injury against MI/R remains unclear.

Hence, dioscin was systematically investigated in a MI/R-injured mouse model *in vivo* and in H9C2 cardiomyocytes *in vitro* subjected to hypoxia/reoxygenation (H/R) injury, presenting the cardioprotective role of dioscin and additionally demonstrating the beneficial function of dioscin against reactive oxygen species, in order to provide new options for the clinical treatment of MI/R.

## 2. Materials and Methods

### 2.1. Animals

Male C57BL/6 mice (22-24 g) were purchased from the Animal Core of Nanjing Medical University (Nanjing, China). The mice were kept with a standard vivarium with free access to food and water. All animal experiments were approved by the National Institutes of Health Guide for the Care and Use of Laboratory Animals, and the protocols used were also consistent with the Animal Ethics Committee of Nanjing Medical University, Nanjing, China (IACUC-2003006).

The myocardial ischemia/reperfusion injury model was produced as previously reported [26]. Briefly, the left anterior descending (LAD) coronary was tied by a slipknot with a 6-0 silk suture. After 30 min of ischemia, the slipknot was released smoothly and gently until a feeling of release was sensed, at which time, the myocardium began reperfusion and was kept for 3 days. Sham-operated control mice underwent the same surgical procedure, except the suture placed under the left coronary artery was not tied.

Dioscin (80 mg/kg/day, Di'ao Group, Chengdu, China) was administrated to mice for 3 days beginning on the day of operation. Briefly, the dioscin was first dissolved in dimethyl sulphoxide (DMSO), and then, 5% sodium carboxymethyl cellulose (CMC-Na) was added to make the volume ratio for 1 : 19. Consequently, the mice were given the dissolution by intragastric administration. The preparation and application of the drug's dissolution should be completed in one day. If precipitation occurred in the preparation process, it could be assisted by heating or ultrasound.

### 2.2. Measurement of Cardiac Function by Echocardiography

Echocardiographic measurements were performed on mice using a VisualSonics Vevo® 2100 Imaging System (VisualSonics, Toronto, Canada) with a 40 MHz MicroScan transducer (model MS-550D) [27]. The M-mode echocardiogram was acquired from the parasternal short axis view of the left ventricle at the midpapillary muscle level. Echocardiographic parameters were calculated using the primary measurements and accompanying software. The echocardiographer was blinded to the genetic identity of the mice for all studies.

### 2.3. Histological Assays

Mouse hearts were dissected out, then fixed in 4% paraformaldehyde (PFA, Electron Microscopy Sciences) overnight. After dehydration through a series of ethanol baths, samples were embedded in paraffin wax. Further, 5 *μ*m thick samples of the heart were obtained to perform hematoxylin and eosin (HE) and Masson trichrome staining. Slides were imaged under a light microscope.

### 2.4. Determination of Enzyme Activities

The detection kits of superoxide dismutase (SOD), catalase (CAT), glutathione (GSH), and glutathione peroxidase (GPx) were purchased from a company (Solarbio, China). The activities of these enzymes in the heart were evaluated following the manufacturer's instructions.

### 2.5. Determination of Reactive Oxygen Species (ROS)

The production of ROS was measured as previously described [28]. The samples of the heart section were incubated with the DHE (Beyotime, China) at 37°C for 30 min, then washed three times with PBS for 5 min, and further costained with DAPI (Beyotime, China). The fluorescence intensity was examined using a confocal scanning microscope, and all images were analyzed using ImageJ software.

### 2.6. Quantitative RT-PCR

Total RNA was isolated using the TRIzol Reagent (Invitrogen) from cell or tissue samples. The mRNA expression levels were determined by quantitative reverse transcription polymerase chain reaction (PCR) using SuperScript II Reverse Transcriptase (Thermo Fisher Scientific Inc.) for reverse transcription and a Power SYBR Green PCR Master Mix (Thermo Fisher Scientific Inc.) for quantitative reverse transcription PCR reaction with PCR primers. The measurable of corresponding genes were detected by real-time PCR Detection System (Bio-Rad) and were analysed by CFX Manager 3.1 software (Bio-Rad). The sequences of primers were as follows: Anp-F: 5′-ACC TCC CGA AGC TAC CTA AGT-3′, Anp-R: 3′-CAA CCT TTT CAA CGG CTC CAA-5′; Bnp-F: 5′-GAG GTC ACT CCT ATC CTC TGG-3′, Bnp-R: 3′-GCC ATT TCC TCC GAC TTT TCT C-5′; *β*-Mhc-F: 5′-GAG GGT GGC TCT CAC ACA TTC-3′, *β*-Mhc-R: 3′-TTG GCC TTC GTA AGC AAA CTG-5′; Sod1-F: 5′-AAC CAG TTG TGT TGT CAG GAC-3′, Sod1-R: 3′-CCA CCA TGT TTC TTA GAG TGA GG-5′; Sod2-F: 5′-CAG ACC TGC CTT ACG ACT ATG G-3′, Sod2-R: 3′-CTC GGT GGC GTT GAG ATT GTT-5′; Cat-F: 5′-AGC GAC CAG ATG AAG CAG TG-3′, Cat-R: 3′-TCC GCT CTC TGT CAA AGT GTG-5′; Nrf2-F: 5′-CCA TTT ACG GAG ACC CAC CGC CTG-3′, Nrf2-R: 3′-CTC GTG TGA GAT GAG CCT CTA AGC GG-5′; Nox2-F: 5′-ACT CCT TGG GTC AGC ACT GG-3′, Nox2-R: 3′-GTT CCT GTC CAG TTG TCT TCG-5′; and 18s-F: 5′-GCC TCC TCC TCC TCT CTC-3′, 18s-R: 3′-GCT ACT GGC AGG ATC AAC C-5′.

### 2.7. Statistical Analysis

Continuous variables that approximated the normal distribution were expressed as mean ± SD. Comparison between groups was subjected to ANOVA followed by the Bonferroni correction for post hoc *t*-test. Data expressed as proportions were assessed with a chi-square test. Two-sided tests have been used throughout, and *P* values < 0.05 were considered statistically significant. GraphPad Prism 8 was used to evaluate data.

## 3. Results

### 3.1. Dioscin Improves Cardiac Dysfunction in MI/R-Injured Mice

**Figure 1:**
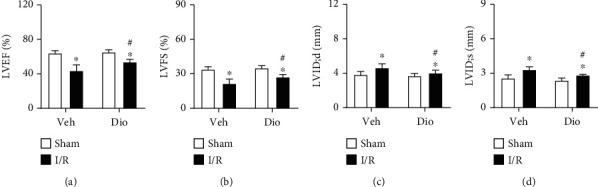
Dioscin improves cardiac function in ischemic/reperfusion mice. The C57BL/6 mice were subjected to myocardial ischemic/reperfusion (MI/R) surgery. Echocardiographic parameters for (a) left ventricular ejection fraction (LVEF, %), (b) left ventricular fractional shortening (LVFS, %), (c) left ventricular end diastolic internal dimension (LVID; d, mm), and (d) left ventricular end systolic internal dimension (LVID; s, mm). *n* = 8 each group. Data are mean ± SD. ^∗^*P* < 0.05 vs. sham group, ^#^*P* < 0.05 vs. I/R group.

In order to explore the protective role of dioscin in response to MI/R injury *in vivo*, mice were subjected to myocardial ischemic/reperfusion surgery. Echocardiography exhibited that the significant increases of cardiac function markers of the left ventricular ejection fraction (LVEF) and the left ventricular fractional shortening (LVFS) were observed in the MI/R with the dioscin treatment group compared to the MI/R group (Figures 1(a) and 1(b)). Furthermore, we found that mice subjected to MI/R treated with dioscin presented improved cardiac function, as evidenced by the preserved left ventricular end diastolic internal dimension (LVID; d) and left ventricular end systolic internal dimension (LVID; s), when compared with vehicle-treated mice (Figures 1(c) and 1(d)).

**Figure 2:**
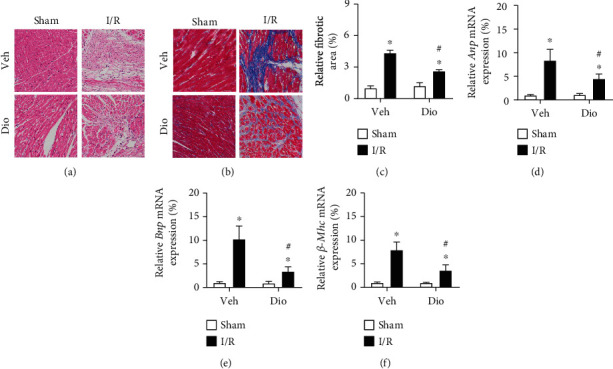
Dioscin alleviates cardiac fibrosis in MI/R injury mice. (a) Representative images of HE staining (bar = 100 *μ*m), *n* = 3 each group. (b) Representative images of Masson trichrome-stained (bar = 100 *μ*m), *n* = 3 each group. (c) The measurement of relative fibrosis area (%). The mRNA expression of (d) *Anp*, (e) *Bnp*, and (f) *β-Mhc*. *n* = 6 each group. Data are mean ± SD. ^∗^*P* < 0.05 vs. sham group, ^#^*P* < 0.05 vs. I/R group.

To examine whether the cardiac fibrosis was prevented by dioscin or not, a series of staining were performed on heart sections. Hematoxylin and eosin (HE) staining revealed widespread myocardial structural disorder, while treatment with dioscin markedly ameliorated histological features in myocardial tissue (Figure 2(a)). Moreover, collagen accumulation in the interstitial space, which was detected by Masson's trichrome staining, increased obviously in the heart sections of the MI/R group, and this increase was improved significantly in the MI/R with treatment dioscin group (Figures 2(b) and 2(c)). We further examined the expression of biomarkers for cardiac function, finding that the expression of natriuretic peptide A (*Anp*), natriuretic peptide B (*Bnp*), and beta-myosin heavy polypeptide cardiac muscle (*β*-*Mhc*) was reduced after being treated with dioscin in MI/R injury hearts compared to the MI/R group (Figures 2(d)–2(f)). These results indicated that dioscin improves cardiac function and alleviates cardiac fibrosis against MI/R injury.

### 3.2. Dioscin Modulates Antioxidant Status in MI/R Mice

**Figure 3:**
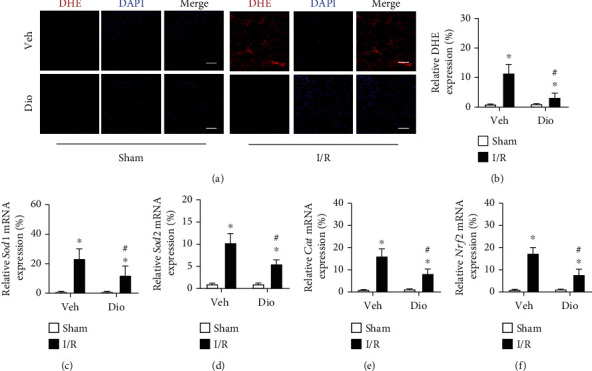
Dioscin changes the oxidant response after I/R. (a) Representative images of double staining of heart sections with dihydroethidium (DHE) (red) and DAPI (blue) (bar = 20 *μ*m), *n* = 3 each group. (b) The quantification of relative expression of reactive oxygen species. The mRNA expression of antioxidant genes, including (c) *Sod1*, (d) *Sod2*, (e) *Cat*, and (f) *Nrf2*. *n* = 6 each group. Data are mean ± SD. ^∗^*P* < 0.05 vs. sham group, ^#^*P* < 0.05 vs. I/R group.

Because of the involvement of ROS in MI/R injury, we detected the ROS levels in perfused hearts. Dihydroethidium (DHE) staining was performed using heart sections, indicating that the levels of ROS in MI/R-induced heart tissues were significantly increased compared with those in the sham group, and dioscin treatment markedly attenuated the elevated production of ROS (Figures 3(a) and 3(b)).

To identify the mechanism underlying how dioscin regulates antioxidant stress in response to MI/R injury, we subsequently detected the mRNA level of corresponding oxidative genes. We found that the expressions of superoxide dismutase 1 (*Sod1*), superoxide dismutase 2 (*Sod2*), catalase (*Cat*), and nuclear factor erythroid 2-related factor 2 (*Nrf2*) were upregulated in hearts from the MI/R group compared to sham mice, which was prevented by dioscin treatment (Figures 3(c)–3(f)). These results might reveal that the upregulated expression of ROS in the period of perfusion causes the increase expression of antioxidant genes, while the production of ROS is eliminated by treatment with dioscin to further suppress the expression of antioxidant genes.

**Figure 4:**
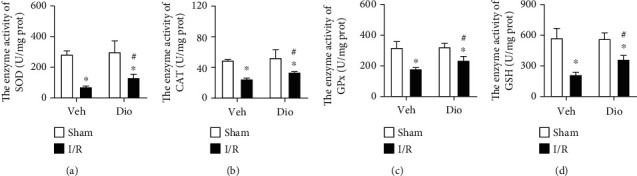
Dioscin modulates antioxidant status in IR hearts. The enzyme activities for (a) SOD, (b) CAT, (c) GPx, and (d) GSH. *n* = 6 each group, repeated twice. Data are mean ± SD. ^∗^*P* < 0.05 vs. sham group, ^#^*P* < 0.05 vs. I/R group.

To further explore the mechanism underlying the protective role of dioscin against MI/R injury, we further measured the activities of antioxidant status-related enzymes. Superoxide dismutase (SOD) is the typical antioxidant enzyme as ROS scavenger. In the MI/R group, the activities of SOD were significant decreased compared to those of sham mice, but the downregulation was prevented by dioscin (Figure 4(a)). What is more, neither catalase (CAT), glutathione (GSH), nor glutathione peroxidase (GPx) plays a protective role in cells from oxidative damage. Dioscin suppressed the declination of MI/R-induced activities of CAT, GSH, and GPx (Figures 4(b)–4(d)). We suspect that the dynamic balance between the antioxidants and oxidant response was damaged by an overburdened high ROS, which further injures these antioxidant enzymes. However, treatment with dioscin recues the decline. Taken together, dioscin could regulate the antioxidant status to repress the production of the ROS level in the MI/R heart.

### 3.3. Dioscin Scavenges ROS in Perfused H9C2 Cells

**Figure 5:**
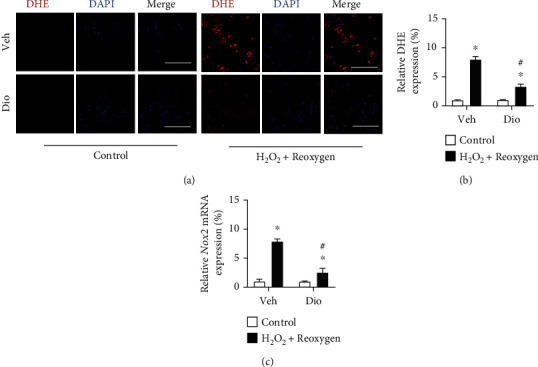
Dioscin abolishes the accumulation of ROS production in H9C2 cells. Representative images of double staining of H9C2 cells with dihydroethidium (DHE) (red) and DAPI (blue) (bar = 50 *μ*m), *n* = 3 each group. (b) The quantification of relative expression of reactive oxygen species. (c) The mRNA expression of *Nox2*. *n* = 6 each group. Data are mean ± SD. ^∗^*P* < 0.05 vs. control group, ^#^*P* < 0.05 vs. H_2_O_2_+reoxygen group.

Considering the results from the in vivo experiments, we further verified whether dioscin abolishes ROS accumulation in myocytes in an oxidant condition. We further performed dihydroethidium (DHE) staining to detect the level of ROS in H_2_O_2_-incubated H9C2 cells, showing that dioscin treatment markedly attenuated the elevated levels of ROS production (Figures 5(a) and 5(b)). Because the nicotinamide adenine dinucleotide phosphate (NADPH) oxidase 2 (*Nox2*) plays a central role in catalyzing the production of superoxide from oxygen, we identified that the expression of *Nox2* was obviously increased in H_2_O_2_-cultured H9C2, and the increase was prevented after dioscin treatment (Figure 5(c)). These results verified that dioscin alleviates perfused injury *via* downregulation of the oxidant response.

## 4. Discussion

In this study, we demonstrated that dioscin, as a natural product, showed a cardioprotective role in response to myocardial ischemic/reperfusion (MI/R) injury. Dioscin has a therapeutic effect *via* downregulation of oxidant stress, reflecting from the elevated levels of the antioxidant enzyme activities, accompanying with the ROS scavenger. These results exhibited that dioscin has potent effects for the treatment of MI/R injury.

Multiple events take part in the pathogenesis of MI/R, including accumulation of ROS, inflammation, perturbation of calcium handing, and metabolic derangements. Considering ROS as the primary cause among these stimuli, the pharmacological antagonists of accumulated succinate sufficiently ameliorated *in vivo* myocardial ischemia/reperfusion injury *via* repressing extensive ROS generation [29]. Further, uric acid aggravates MI/R-induced activation of the NOD-like receptor pyrin domain-containing protein 3 (NLRP3) inflammatory cascade and pyroptosis by promoting ROS generation, while inflammasome inhibitors and ROS scavengers partly reverse the injury [30]. What is more, ROS scavenger N-acetyl cysteine (NAC) was able to reduce the amount of ROS and prevent cell death [31]. Herein, we identified that dioscin was considered as the ROS scavenger in the process of MI/R injury.

Dioscin plays a beneficial role in cardiac protection *via* reducing the production of ROS. MI/R injury is mediated by the elevated production of ROS, which occurs particularly at reperfusion [32]. The nicotinamide adenine dinucleotide phosphate (NADPH) oxidase family is considered as one major source of ROS in cardiomyocytes [33]. Meanwhile, many antioxidant enzymes participate in the elimination of ROS in response to MI/R injury [34]. Additionally, it was reported that dioscin alleviated doxorubicin-induced cardiotoxicity through modulating miR-140-5p-mediated myocardial oxidative stress [35]. Furthermore, dioscin has renoprotective effects against fructose-induced renal damage *via* adjusting sirtuin 3-mediated oxidative stress [36]. And dioscin significantly protected against renal damage by decreasing blood urea nitrogen and creatinine levels and reversing oxidative stress [37].

Summarily, dioscin ameliorates myocardial ischemia/reperfusion injury through suppressing reactive oxygen species *via* downregulation of Nox2 and upregulation of the antioxidative enzyme, including SOD, CAT, GPx, and GSH, leading to alleviate cardiac dysfunction. Our results indicated that dioscin, providing a potential therapeutic strategy, would be beneficial for myocardial ischemic/reperfusion injury.

## Data Availability

The data used to support the findings of this study are available from the corresponding authors upon request.
